# Molecular docking analysis of novel Non-Nucleoside Reverse Transcriptase Inhibitors in development: implication for rational drug design

**DOI:** 10.1186/1742-4690-8-S2-P82

**Published:** 2011-10-03

**Authors:** Udaya Pratap Singh, Ramendra K Singh

**Affiliations:** 1 Department of Pharmaceutical Sciences, Sam Higginbottom Institute of Agriculture Technology & Sciences, (Formerly Allahabad Agricultural Institute) Deemed-to-be-University, Allahabad, India; 2 Nucleic Acids Research Laboratory, Department of Chemistry, University of Allahabad, Allahabad, India

## Background

Human immunodeficiency virus (HIV) from a family of retroviruses is a causative organism for acquired immuno deficiency syndrome (AIDS), affecting more than 40 million people worldwide [1]. Reverse transcription of the HIV single-stranded RNA genome into double-stranded DNA is one of the essential key step in the virus replication life-cycle and acts as lucrative target for drug action. Till date, out of 20 anti-HIV drugs approved for the treatment of HIV infection, eleven approved anti-HIV drugs target RT, and encompass three classes of agents that inhibit RT by different mechanisms (**N**ucleoside **R**everse **T**ranscriptase **I**nhibitors, NRTIs; **N**on-**N**ucleoside **R**everse **T**ranscriptase **I**nhibitors, NNRTIs; **N**ucleotide **R**everse **T**ranscriptase **I**nhibitors, NtRTIs) [2]. The NNRTIs serve as an increasingly important role in the therapy of HIV infection, due to their unique antiviral potency, high specificity and low toxicity. Present work illustrates the probable binding affinity, interatomic contacts and orientation of novel of NNRTIs in development at the allosteric site of the HIV-RT through an iterative computational process and molecular docking to explicate the necessary structural requirement for rational drug design.

## Material and methods

Six NNRTIs in development were selected for the study viz. Etravirine, Rilpivirine, Calanolide A, IDX899, RDEA806 and Lersivirine). These selected molecules were rigorously analyzed through Lipinski’s Rule of 5 (Ro5) by molinspiration software [3]. In an order to explicate the binding affinity, interatomic contacts and orientation of NNRTIs at the active of HIV-RT, a molecular docking study were carried out by utilizing scoring function such as PLP1, PLP2, Jain, PMF, Lig_Internal_Energy, binding energy, and ultimately dock score. Docking study was performed by using the LigandFit as docking module within package of Discovery Studio 2.5. Position optimization of both ligand and proteins were performed by using all-atom CHARMm forcefield with Adopted Basis set Newton Raphson minimization algorithm until the root mean square (r.m.s) gradient for potential energy was less than 0.05 kcal/mol/Å. The cavity within a receptor defined as active site and an input site sphere was defined over the active site with a radius of 5 Å.

## Results

All six molecules successfully passed the Lipinski’s Ro5 filter to be as a probable drug candidate except some minor violations. In terms of molecular docking, they presented excellent scoring and binding affinity towards the receptor due to the close interatomic contacts through strong H-bonds Lys103 (Calanolide A), His235, Tyr318 (RDEA806), Lys101 (Rilpivirine, Etravirine) Val179 (Etravirine), pi-pi Tyr181 (IDX899, RDEA806, Rilpivirine, Lersivirine) and pi-cation interactions through Lys101 (RDEA806), Tyr103 (Rilpivirine).

## Conclusion

As a concluding remark, preliminary screening by Lipinski’s Ro5 acted as filter to obtain potential molecule from large datasets, whereas, pi-pi interaction was coined as a major interatomic interaction for stabilizing receptor-ligand complex and minor with hydrogen bonding and pi-cation interaction. We have anticipated a pivotal facet for designing the next generation of NNRTIs with improved activity based upon molecular docking studies.
